# Valosin-containing protein-regulated endoplasmic reticulum stress causes NOD2-dependent inflammatory responses

**DOI:** 10.1038/s41598-022-07804-1

**Published:** 2022-03-10

**Authors:** Maryam Ghalandary, Yue Li, Thomas Fröhlich, Thomas Magg, Yanshan Liu, Meino Rohlfs, Sebastian Hollizeck, Raffaele Conca, Tobias Schwerd, Holm H. Uhlig, Philip Bufler, Sibylle Koletzko, Aleixo M. Muise, Scott B. Snapper, Fabian Hauck, Christoph Klein, Daniel Kotlarz

**Affiliations:** 1 Dr. von Hauner Children’s Hospital, Department of Pediatrics, University Hospital, Ludwig-Maximilians-Universität (LMU) Munich, 80337 Munich, Germany; 2 Laboratory for Functional Genome Analysis (LAFUGA), Gene Center, LMU Munich, Munich, Germany; 3 Translational Gastroenterology Unit and Department of Pediatrics, and Biomedical Research Centre, University of Oxford, Oxford, UK; 4 Department of Pediatric Gastroenterology, Nephrology and Metabolic Diseases, Charité Universitätsmedizin Berlin, Corporate Member of Freie Universität Berlin, Humboldt-Universität Zu Berlin, Berlin, Germany; 5 Department of Pediatrics, School of Medicine Collegium, Medicum University of Warmia and Mazury, Olsztyn, Poland; 6 SickKids Inflammatory Bowel Disease Center, Research Institute, Hospital for Sick Children, Toronto, ON M5G1X8 Canada; 7 Cell Biology Program, Research Institute, Hospital for Sick Children, Toronto, ON M5G1X8 Canada; 8 VEO-IBD Consortium, University Hospital, LMU Munich, 80337 Munich, Germany; 9 Division of Gastroenterology, Hepatology and Nutrition, Department of Pediatrics, Hospital for Sick Children, University of Toronto, Toronto, ON M5G1X8 Canada; 10 Department of Biochemistry, University of Toronto, Toronto, ON M5G1A8 Canada; 11 Division of Gastroenterology, Hepatology and Nutrition, Boston Children’s Hospital, Boston, MA 02115 USA; 12 Department of Medicine, Harvard Medical School, Boston, MA 02115 USA; 13 Division of Gastroenterology, Brigham and Women’s Hospital, Boston, MA 02115 USA; 14 Gene Center, LMU Munich, Munich, Germany; 15 Deutsche Zentrum für Infektionsforschung (DZIF), Inhoffenstraße 7, 38124 Braunschweig, Germany

**Keywords:** Immunogenetics, Gastrointestinal diseases, Genetics

## Abstract

NOD2 polymorphisms may affect sensing of the bacterial muramyl dipeptide (MDP) and trigger perturbed inflammatory responses. Genetic screening of a patient with immunodeficiency and enteropathy revealed a rare homozygous missense mutation in the first CARD domain of NOD2 (ENST00000300589; c.160G > A, p.E54K). Biochemical assays confirmed impaired NOD2-dependent signaling and proinflammatory cytokine production in patient’s cells and heterologous cellular models with overexpression of the NOD2 mutant. Immunoprecipitation-coupled mass spectrometry unveiled the ATPase valosin-containing protein (VCP) as novel interaction partner of wildtype NOD2, while the binding to the NOD2 variant p.E54K was abrogated. Knockdown of VCP in coloncarcinoma cells led to impaired NF-κB activity and *IL8* expression upon MDP stimulation. In contrast, tunicamycin-induced ER stress resulted in increased *IL8, CXCL1,* and *CXCL2* production in cells with knockdown of VCP, while enhanced expression of these proinflammatory molecules was abolished upon knockout of NOD2. Taken together, these data suggest that VCP-mediated inflammatory responses upon ER stress are NOD2-dependent.

## Introduction

The innate immune system has crucial functions in detection and eradication of pathogens. The recognition of microbial-associated molecular patterns (MAMPs) and induction of inflammatory responses depends on specific pattern recognition receptors (PRRs)^1^. The intracellular PRR nucleotide-binding oligomerization domain protein 2 (NOD2) senses muramyl dipeptide (MDP), an evolutionary conserved component of the bacterial cell walls^2^. Upon MDP challenge, intracellular NOD2 oligomerizes and recruits receptor-interacting serine/threonine-protein kinase 2 (RIPK2) through CARD-CARD homotypic interaction leading to activation of downstream signaling pathways such as nuclear factor-κB (NF-κB) and mitogen-activated protein kinase (MAPK) signaling^3–7^. Dysregulated NOD2 signaling has been implicated in several inflammatory disorders such as Blau syndrome, sarcoidosis, allergy, asthma and autoimmunity^8–10^. In particular, *NOD2* has been recognized as the key susceptibility gene in Crohn’s disease (CD)^8^. NOD2 polymorphisms associated with CD are mainly located in the leucine-rich repeat domain which is responsible for ligand sensing and binding.

Here, we report a rare missense mutation affecting the first CARD domain of NOD2 that has been identified in a child with enteropathy and was associated with defective MDP-dependent signaling, abrogated interaction with RIPK2, as well as impaired cytokine responses. The characterization of this rare mutation unveiled Valosin-containing protein (VCP) as novel interaction partner of NOD2. VCP is a ubiquitously expressed ATPase with pleiotropic functions in controlling Ubiquitin-proteasome system (UPS)-mediated protein degradation in endoplasmic reticulum (ER)-associated protein degradation (ERAD), apoptosis, and autophagy^11–13^. Our study highlighted that VCP-mediated proinflammatory responses during ER stress are NOD2-dependent.

## Results

### Defective NOD2 signaling caused by a biallelic germline mutation affecting the first CARD domain

**Figure 1:**
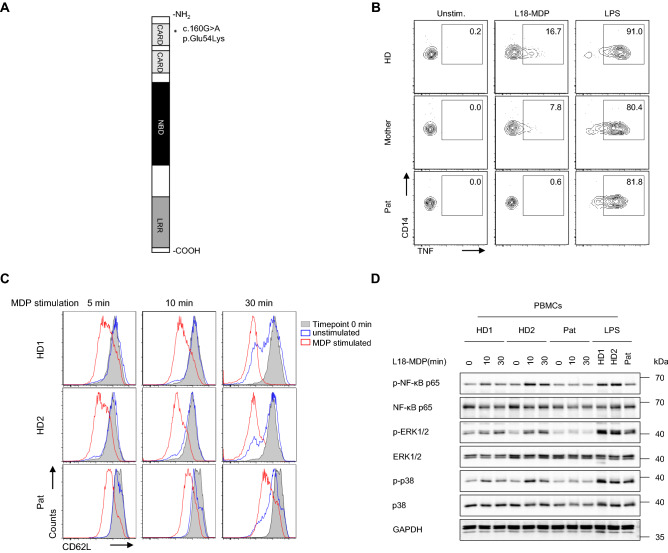
Defective NOD2-mediated signaling in patient primary cells. (**A**) Schematic illustration of NOD2 protein domains. The mutation identified in patient is depicted by an asterisk. (**B**) Representative FACS analysis of TNF staining (n = 3) on PBMC-derived monocytes (CD14^+^) isolated from patient (Pat), mother and a healthy donor (HD) and stimulated with L18-MDP or LPS. (**C**) Representative FACS analysis of CD62L expression (n = 3) on neutrophils isolated from patient (Pat) and two healthy donors (HD) upon L18-MDP stimulation. (**D**) Representative immunoblotting of serum-starved PBMCs from patient (Pat) and two healthy donors (HD) stimulated with L18-MDP or LPS.

Whole exome sequencing was conducted to elucidate the genetic etiology in a one-year-old female patient presenting with intractable diarrhea, recurrent perianal candida dermatitis, hemophagocytic lymphohystiocytosis (HLH), and prolonged Norovirus infection. Genetic screening revealed rare homozygous missense mutations in *NOD2* (ENST00000300589; c.160G > A, p.E54K) and *STXBP2* (ENST00000441779; c.949C > G, p.L317V). Even though the variant in *STXBP2* was predicted to be benign, this mutation likely has caused HLH associated with impaired NK cell degranulation (Supplementary Fig. 1). By contrast, the *NOD2* missense mutation has been proposed as deleterious based on PolyPhen and SIFT and thus, may contribute to the gastrointestinal phenotypes (intractable diarrhea, recurrent perianal dermatitis). Our study was not specifically designed to address whether the *NOD2* variant was the causal or risk factor of disease in our patient, but the distinct location of the variant triggered assessment of NOD2-mediated signaling and interacting networks, since previously reported CD-associated *NOD2* variants are mainly localized in or near the LRR domain (Fig. 1A)^14–16^.

**Figure 2:**
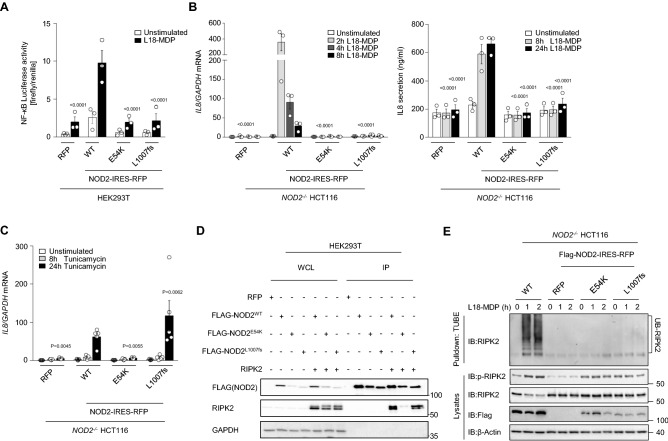
The NOD2 p.E54K variant leads to abrogation of both PGN-dependent and -independent signaling. (**A**) NF-kB luciferase reporter activity upon challenge with L18-MDP (7 h) in HEK293T cells overexpressing NOD2 wild-type (WT) or indicated mutants. (**B** and **C**) Quantitative RT–PCR analysis and ELISA of *IL8* expression upon stimulation with L18-MDP (**B**) or tunicamycin (**C**) in heterologous HCT116 cells. (**D**) Representative immunoprecipitation of FLAG-tagged NOD2 (n = 3) on HEK293T cells that were transiently transfected with Flag-NOD2 WT or indicated mutants alone or along with WT RIPK2. (**E**) Representative TUBE assay (n = 2) from L18-MDP-treated heterologous HCT116 cells. Data represent mean ± SEM of three (**A** and **B**) or five (**C**) independent experiments. P values for each treatment group are calculated in comparison to WT. WCL, whole cell lysate; IP, Immunoprecipitates.

To elucidate the effects of the identified NOD2 mutation on the canonical NOD2-mediated signaling, we assessed intracytoplasmic TNF production in patient’s peripheral blood mononuclear cells (PBMC)-derived monocytes upon stimulation with the ligand L18-MDP. While patient-derived monocytes showed a normal response to lipopolysaccharide (LPS), TNF expression was reduced in L18-MDP-treated cells as compared with healthy donors (Fig. 1B). In addition, patient-derived neutrophils showed reduced CD62L shedding upon MDP stimulation (Fig. 1C), confirming defective NOD2 signaling in patient innate immune cells. Correspondingly, patient’s PBMCs revealed reduced phosphorylation of NF-κB p65, ERK1/2, and/or p38 upon stimulation with L18-MDP (Fig. 1D). Furthermore, luciferase reporter assays on HEK293T cells ectopically expressing the NOD2 variant p.E54K showed impaired NF-κB activity in response to L18-MDP comparable with the NOD2 variant p.L1007fsX1008 that has previously been associated with CD^15^ (Fig. 2A).

To gain insights into the pathomechanisms of the mutation in the context of intestinal inflammation, we engineered coloncarcinoma-derived HCT116 cells with a CRISPR/Cas9-mediated NOD2 knockout (KO) and subsequent lentiviral overexpression of wild-type (WT) or mutant (p.E54K and p.L1007fsX1008) NOD2 variants. In contrast to WT reconstitution, HCT116 cells with NOD2 mutants showed reduced expression and secretion of IL-8 upon treatment with L18-MDP (Fig. 2B).

Emerging evidence highlights that NOD2 has critical functions apart from peptidoglycan (PGN) sensing. Notably, NOD2 has been implicated in mediating proinflammatory responses triggered by ER stress^17^. To assess the PGN-independent functions of NOD2, we evaluated the expression of the proinflammatory cytokine *IL8* in engineered HCT116 cells upon ER stress. Treatment with tunicamycin resulted in impaired *IL8* production in cells with expression of the NOD2 variant p.E54K as compared with WT NOD2 reconstituted cells. Interestingly, we could detect normal expression of *IL8* for the NOD2 variant p.L1007fsX1008 suggesting genotype-specific mechanisms of ER stress-induced proinflammation in the context of NOD2 deficiency (Fig. 2C).

### Impaired RIPK2 binding and ubiquitination by the NOD2 mutant p.E54K

NOD2 is critical in mediating inflammatory signaling pathways in response to invading pathogens via the interaction of RIPK2 with its CARD domain^6^. Previously reported NOD2 polymorphisms associated with CD are mainly localized in the LRR domain^14,18–25^, however two heterozygous variants (p.R38M and p.R138Q) affecting the first and second CARD domain have been suggested to alter RIPK2 recruitment and NF-κB signaling^26^. To assess whether the NOD2 variant p.E54K affects interaction with RIPK2, we conducted co-immunoprecipitation experiments using anti-FLAG beads in HEK293T cells ectopically expressing FLAG-tagged NOD2 WT or mutants along with WT RIPK2. While the NOD2 variant p.L1007fsX1008 showed normal binding to RIPK2, the interaction of the mutant p.E54K and RIPK2 was significantly reduced (Fig. 2D).

Previous studies have demonstrated that polyubiquitination and autophosphorylation of RIPK2 is triggered upon NOD2 activation^4,7,27,28^. Despite the different abilities of the NOD2 mutants p.E54K and p.L1007fsX1008 to interact with RIPK2, we could detect reduced phosphorylation of RIPK2 at position S176 by immunoblotting as well as impaired ubiquitination of RIPK2 by employing tandem ubiquitin-binding entities upon MDP stimulation in both NOD2 variants (Fig. 2E)*.* These data suggest that defective polyubiquitination and/or autophosphorylation of RIPK2 may represent an underlying common mechanism for reduced MDP-dependent signaling associated with NOD2 polymorphisms.

### Identification of VCP as novel interaction partner of NOD2

**Figure 3:**
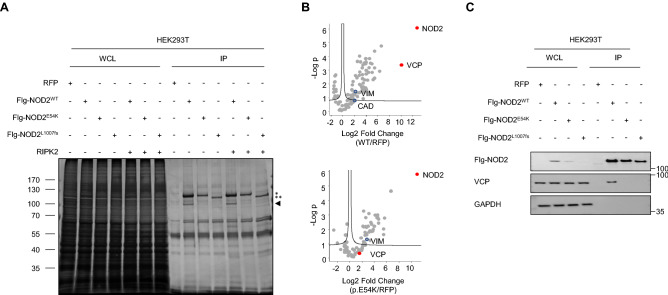
Identification of VCP as novel interaction partner of NOD2. (**A**) Representative SDS-PAGE and silver staining (n = 3) of cell lysates from immunoprecipitation experiments on HEK293T cells that ectopically expressed Flag-NOD2 WT (WT) or mutant alone or along with WT RIPK2. While the asterisks indicate expression of Flag-tagged NOD2 proteins (* full length, ** truncated), the arrow points to an interaction protein of Flag-NOD2 WT (molecular weight about 100 kDa) that was not detectable in the NOD2 mutants p.E54K and p.L1007fsX1008. (**B**) Volcano plots of proteins enriched in NOD2 WT or the mutant (p.E54K) versus the RFP control. (**C**) Flag IP on HEK293T cells transiently transfected with Flag-NOD2 WT or mutants (n = 3). WCL, whole cell lysate. IP, Immunoprecipitates.

To study the altered interactome of the NOD2 variant p.E54K, cell lysates from immunoprecipitation experiments on HEK293T cells with ectopic expression of WT or mutant NOD2 were subjected to SDS-PAGE. Silver staining revealed a band with a molecular weight of about 100 kDa only present in cells overexpressing WT NOD2 (Fig. 3A). Using co-immunoprecipitation and nano liquid chromatography tandem mass spectrometry (LC–MS/MS), we identified VCP as novel NOD2 interacting protein enriched in cells reconstituted with WT NOD2. In contrast, cells with expression of the variants p.E54K or p.L1007fsX1008 showed abrogated interaction of VCP with NOD2 (Fig. 3B and C). Notably, we also identified vimentin (VIM) and carbamoyl phosphate synthetase/aspartate transcarbamylase/dihydroorotase (CAD) among the list of known NOD2 interacting proteins; thus increasing the confide in our screening approach. While vimentin was enriched in both NOD2 WT and p.E54K expressing cells as compared with RFP controls, CAD was found to be enriched only in cells overexpressing WT NOD2 (Fig. 3B). Finally, the interaction of endogenous VCP/NOD2 proteins in HCT116 cells was confirmed by immunoprecipitation with an antibody binding to VCP and co-precipitation of NOD2 (Supplementary Fig. 2).

VCP is an evolutionarily conserved AAA + ATPase governing diverse biological functions, in particular in the UPS and ER-associated degradation (ERAD)^29^. VCP mutations have been associated with several diseases such as myopathy, Paget’s disease, dementia, amyotrophic lateral sclerosis and Huntington’s disease^29,30^. However, the exact role of VCP in health and disease remains elusive. Previously, VCP has been listed as potential NOD2 interaction partner in one of three yeast two hybrid (Y2H) screens^31^ and proteomic studies have revealed VCP in the group of proteins that are differentially expressed in HEK293T cells overexpressing the NOD2 variant p.L1007fsX1008^32^. These studies support our findings that VCP is an interaction partner of NOD2 but did not provide any functional links for the regulatory role of VCP in NOD2 signaling.

### VCP controlled ER stress causes inflammatory responses in a NOD2-dependent manner

**Figure 4:**
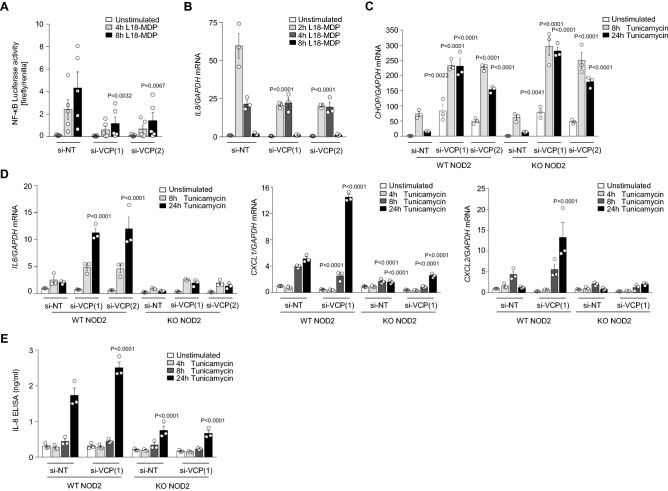
VCP-mediated ER-stress-induced proinflammatory responses are NOD2-dependent. (**A**) NF-kB-sensitive luciferase reporter activity (n = 5) and (**B**) quantitative RT–PCR analysis of *IL8* production in L18-MDP-treated HCT116 cells transfected with si-NT (non-targeting control) or si-VCP (see also Figure S4). (**C** and **D**) Quantitative RT–PCR analysis of *CHOP* (**C**) and *IL8*, *CXCL1*, *CXCL2* and *TNF* (**D**) transcriptional level on HCT116 cells transfected with si-NT or si-VCP upon tunicamycin stimulation (see also Figure S6). (**E**) ELISA of IL-8 secretion upon tunicamycin treatment in WT and NOD2 KO cells. (**B**, **C**, **D** and **E**) Data represent mean ± SEM of three independent experiments. P values in each treatment group were calculated in comparison to non-targeting control.

To study VCP function in the context of NOD2 signaling, we engineered heterologous HCT116 cells with siRNA-mediated knockdown of VCP and evaluated MDP-induced NF-κB activity and *IL8* expression. Luciferase reporter assays showed impaired NF-κB activation in cells with knockdown of VCP in response to L18-MDP treatment (Fig. 4A). Correspondingly, we could detect decreased expression of *IL8* in MDP-treated cells upon VCP knockdown, as compared with cells transfected with non-targeting siRNA (Fig. 4B). However, we could not observe a direct effect of VCP knockdown on phosphorylation of RIPK2 (S176) or binding of RIPK2 to NOD2 (Supplementary Fig. 3).

VCP plays a critical role in controlling UPR and inhibition of VCP resulted in increased ER stress^33^. Moreover, enhanced ER stress and activated UPR in intestinal epithelial cells have been reported in patients with CD and ulcerative colitis (UC)^34^. Recently, NOD1 and NOD2 have been proposed as molecular bridges linking ER stress to proinflammatory responses^17^. To examine the influence of NOD2 on VCP-mediated ER stress functions, we transfected HCT116 cells with NOD2 KO or lentiviral reconstitution of NOD2 WT with siRNAs targeting VCP. Knockdown efficiency was assessed by qPCR measurement of VCP mRNA as well as immunoblotting of VCP protein (Supplementary Figs. 4 and 5). Using this heterologous cellular model, we could confirm previous findings that VCP knockdown induces increased UPR, as demonstrated by enhanced expression of C/EBP homologous protein (CHOP) (Fig. 4C) as well as increased phosphorylation of PERK and eIF2α, while splicing of XBP1 appeared unaffected (Supplementary Fig. 5). Whereas additional knockout of NOD2 resulted in slight increase of the activated PERK-eIF2α axis (Supplementary Fig. 5), we could not observe a significant upregulation of the transcription of CHOP, which is a downstream target modulated by all three signaling branches of UPR (Fig. 4C). Moreover, treatment with tunicamcyin in NOD2 WT reconstituted cells induced enhanced *IL8*, *CXCL1,* and *CXCL2* expression upon knockdown of VCP, while increased proinflammatory cytokine and chemokine expression could not be observed in NOD2 KO cells (Fig. 4D and E). Differences in *IL8*, *CXCL1,* and *CXCL2* expression between NOD2 knockout and WT reconstituted cells were not associated with increased cell death (Supplementary Fig. 6). Taken together, these findings suggest that inflammatory responses caused by VCP-regulated ER stress are NOD2-dependent.

## Discussion

NOD2 is a key receptor of innate immunity and the first genetic locus that has been associated with inflammatory bowel disease (IBD)^14,15^. Since most CD-associated NOD2 variants are located in the LRR domain, the identification of a biallelic missense mutation affecting the CARD domain of NOD2 in a patient with enteropathy prompted us to investigate the signaling and interaction network of the mutant NOD2 protein in greater detail. Even though the HLH-associated phenotype in our patient is likely caused by the *STXBP2* mutation, the *NOD2* sequence variant may be a risk factor contributing to the gastrointestinal phenotypes (intractable diarrhea, recurrent perianal dermatitis). The function of NOD2 in pathogen recognition or peptidoglycan sensing has been previously acknowledged^35,36^, however the role of NOD2 during ER stress remains still elusive. Our biochemical study showed impaired NOD2-governed PGN-dependent and independent signaling in primary patient cells as well as cellular models and unveiled VCP as novel interaction partner of NOD2 that regulates ER stress-mediated inflammatory responses.

Several studies have suggested the implication of dysregulated UPR in different inflammatory conditions such as neurodegenerative diseases and IBD^37–40^. A growing body of evidence indicate reciprocal relationships between inflammation and ER stress^41^. While inflammatory stimuli like pattern-recognition receptor (PRR) ligands or ROS can induce UPR, activation of the three main UPR pathways can trigger NF-κB- and MAPK-dependent inflammatory responses leading to the expression of proinflammatory cytokines such as IL-6 and TNF^17,42–44^. In the context of intestinal inflammation, mice with KO of *Ire1* and *Xbp* have been shown to have increased sensitivity to dextran sodium sulfate (DSS)-induced colitis^37,38^.

NOD2 has been previously implicated in regulating ER stress^17^. For example, Laccase domain containing-1 (LACC1)-dependent induction of ER stress has been documented in macrophages upon MDP stimulation^45^. Furthermore, previous studies have shown that NOD1/NOD2/RIPK2-dependent inflammation can be triggered by ER stress in mouse bone-marrow-derived macrophages (BMDMs) via the IRE1α/TRAF2 pathway^17^. Recently, Pham et al. showed that mice lacking NOD1 and NOD2 or RIPK2 exhibit increased systemic bacterial burdens after infection with *Chlamydia* suggesting a relevant NOD2-dependent link between ER stress and bacteria-specific inflammatory responses^46^. However, the exact mechanisms of NOD2 activation and function during ER stress still remain largely unknown. Our study on NOD2-deficient epithelial cells suggested that the identified NOD2 germline mutation affecting the CARD domain showed compromised pro-inflammatory responses upon tunicamycin-induced ER stress. Interestingly, we could observe genotype-specific phenotypes, since overexpression of the NOD2 variant p.E54K in NOD2 knockout HCT116 cells resulted in altered *IL8* expression in comparison to cells expressing the NOD2 variant p.L1007fsX1008. Recently, Pei et at. have shown that NOD2 mediates proinflammatory responses upon different types of UPR via interaction of its nucleotide binding domains with sphingosine-1-phosphate^47^. Corresponding to their finding that ER stress activated NOD2 independent of the LRR domain, we could observe comparable transcriptional level of *IL8* in cells overexpressing the NOD2 p.L1007fsX1008 variant and WT NOD2. Thus, our investigation on a rare sequence indicated altered ER stress as possible mechanism how NOD2 polymorphisms may contribute to disease development and behavior.

The CARD domain of NOD2 is known to be important for the interaction with the adaptor protein RIPK2^48^. Our study revealed impaired RIPK2 interaction for the identified mutation p.E54K affecting the CARD domain but not the LRR domain variant p.L1007fsX1008. However, we could observe reduced phosphorylation and abrogated ubiquitination of RIPK2 as a potential common pathomechanism for the impaired MDP-triggered NF-κB activity in NOD2-deficient cells. Consistently, X-linked Inhibitor of Apoptosis (XIAP) E3 ubiquitin ligase activity mediating ubiquitination of RIPK2 has been previously reported to be indispensable for NF-κB activation initiated by NOD2 stimulation^49^. The relevance of this signaling axis for human disease has been demonstrated by loss-of-function XIAP mutations causing a severe immunodeficiency disorder^50,51^. In routine diagnostics, analysis of defective MDP-dependent NOD2 signaling is used to determine XIAP deficiency^52^. Since both mutations exhibited abrogated RIPK2 ubiquitination, future studies investigating the recruitment of XIAP to the NOD2 complex might provide further insights on the pathomechanisms of the NOD2 variants.

In view of the impaired RIPK2 interaction and posttranslational modification, we sought to decipher the interaction network of the NOD2 variant p.E54K. Using an immunoprecipitation-coupled mass spectrometry screen, we identified VCP as a novel NOD2 interaction partner that was associated with wild-type NOD2 protein but not with the NOD2 variants p.E54K and p.L1007fsX1008. VCP is an abundant ubiquitin-dependent ATPase that implicates in myriad of cellular processes such as ERAD, autophagy, DNA damage response, apoptosis and ubiquitin–proteasome-dependent protein degradation^11–13,53,54^. Heterozygous germline mutations in *VCP* have been previously associated with Paget disease of bone and frontotemporal dementia, amyotrophic lateral sclerosis (ALS) and type 2 Charcot–Marie–Tooth disease^55–57^. Furthermore, increased level of proinflammatory cytokines have been observed in the plasma and myoblasts of patients with VCP mutations^58^. The function of VCP in the ERAD pathway has been reported to be regulated through interaction with the deubiquitinase ATAXIN3^59^. Interestingly phosphorylation of ATAXIN3 by NOD2 and TLR2 in myeloid cells has been recently shown to mediate mitochondrial reactive oxygen species production and bacterial clearance^60^. To evaluate plausible functions of VCP in NOD2 signaling, we used VCP-silenced cellular models that were stimulated with the NOD2 canonical stimuli MDP. Consistent with previous studies demonstrating impaired TNF- and IL-1β-triggered NF-κB signaling in VCP-deficient cells^61^, we observed impaired NF-κB activity and proinflammatory cytokine responses in cells with knockdown of VCP upon MDP treatment comparable to NOD2-deficient cells. Strikingly, our data unveiled VCP as a negative regulator of NOD2 activity during tunicamycin-induced ER stress, as VCP silencing resulted in NOD2-dependent hyperinflammatory responses. While VCP-dependent CHOP transcription was not affected by knockout of NOD2, expression of the members of the CXC family of chemokines *IL8* (CXCL8)*, CXCL1, and CXCL2* was increased in a NOD2-dependent manner. These chemokines have been shown to be important in the regulation of neutrophil activation and migration as well as the induction of exaggerated angiogenesis at sites of inflammation^62^. Notably, the expression and activity of these molecules were positively correlated with the grade of inflammation in IBD patients^63–66^. Thus, altered NOD2-dependent proinflammatory cytokine responses upon ER stress may present a new link in the context of intestinal inflammation, however the exact mechanisms how VCP acts on NOD2 signaling remains elusive. Our data suggested that knockdown of VCP alters UPR but does not directly affect phosphorylation of RIPK2 or binding of RIPK2 to NOD2. Therefore, we propose that VCP-regulated UPR can be sensed by NOD2 and can trigger inflammatory responses in a NOD2-dependent manner. Previous studies have suggested that activation of NF-κB signaling is mediating ER stress-derived inflammation, however we could not observe substantial alteration of NF-κB p65 phosphorylation upon tunicamycin stimulation in VCP-silenced cells (data not shown). Further studies are required to profile ER stress-induced NOD2-dependent proinflammatory responses in greater detail and to decipher the underlying molecular mechanisms.

Taken together, molecular characterization of a rare germline mutation affecting the first CARD domain of NOD2 unveils VCP as novel interaction partner. Functional studies show that VCP controlled ER stress induces inflammatory responses in a NOD2-dependent fashion; thus, providing a new potential mechanistic link and therapeutic target in NOD2-related intestinal inflammation.

## Methods

### Patient

Written informed consent was obtained from the patient, first-degree relatives, and healthy donors for the collection of peripheral blood. The investigation was approved by the respective institutional review boards of the LMU Munich and conducted in accordance with current ethical and legal frameworks.

### DNA sequencing

Next-generation sequencing was performed at the Dr. von Hauner Children's Hospital NGS facility. Genomic DNA was isolated from whole blood (Qiagen) for the generation of whole-exome libraries using the SureSelect XT Human All Exon V6 + UTR kit (Agilent Technologies). Barcoded libraries were sequenced with a NextSeq 500 platform (Illumina) to an average coverage depth of 90x. Bioinformatics analysis used Burrows-Wheeler Aligner (BWA 0.7.15), Genome Analysis ToolKit (GATK 3.6) and Variant Effect Predictor (VEP 89). The frequency filtering used allele frequencies from public (e.g. ExAC, GnomAD and GME) and in house databases. The potentially causative variants were confirmed by Sanger sequencing for the patient and informative family members.

### Plasmids and retroviral-mediated gene expression

Full-length human WT *NOD2* was amplified from healthy donor (HD) cDNA. Patient-specific mutations (encoding p.E54K and P.L1007fsX1008) were introduced by site-directed PCR mutagenesis. WT and mutated *NOD2* cDNAs or fusion constructs with an N-terminal FLAG-tag were cloned into the IRES-EGFP or IRES-RFP bicistronic lentiviral pRRL vectors. Lentiviral particles were produced by transfection of HEK293T cells with viral packaging plasmids (psPAX2 and pMD2.G, kindly provided by Didier Trono, Geneva) together with lentiviral pRRL vectors enconding NOD2 WT or mutants. Supernatants were collected every 24 h for 3 days and filtratered prior to transduction of NOD2-deficient HCT116 cells in the presence of 8 µg/ml polybrene (Sigma-Aldrich). Sorting of transduced cells was conducted on a BD FACSAria cell sorter (BD Bioscience) based on RFP or EGFP mean fluorescence intensity. Human WT *RIPK2* was amplified from the verified cDNA sequence clone (GE Dharmacon, cat.no. MHS6278-202830678) and cloned into the pRRL-IRES-RFP plasmid.

### Antibodies and reagents

Antibodies for phospho-NF-κB (p65) (Ser536) (3033, clone number 93H1), NF-κB (p65) (8242, clone number D14E12), phospho-p44/42 MAPK (Erk1/2) (Thr202/Tyr204) (4370, clone number D13.14.4E), p44/42 MAPK (Erk1/2) (9102), phospho-p38 MAPK (Thr180/Tyr182) (4511, clone number D3F9), p38 MAPK (9212), RIP2 (4142, clone number D10B11), phospho-RIP2 (Ser176) (14397S), VCP (2648), XBP-1S (12782 s), phospho-eIF2α (Ser51) (3597 s), and HRP-conjugated anti-rabbit IgG (7074) were purchased from Cell Signaling Technology. Phospho-PERK (T982) (ab192591) was purchased from Abcam. Beta-Actin-HRP (sc-47778, clone number c4) and GAPDH (sc-47724, clone number 0411) were purchased from Santa Cruz Biotechnology. Anti-Flag antibody (F1804, clone number M2), Anti-FLAG M2 Affinity Gel (A2220), lipopolysaccharide (LPS) (L2654) and tunicamycin Streptomyces sp. (T7765) were procured from Sigma-Aldrich. HRP-conjugated goat anti-mouse IgG (554002), PE mouse anti-human TNF (559321, clone number MAb11) and anti-CD14-FITC (557153, clone M5E2), anti-CD14–BV786 (563698, clone M5E2), anti-CD3-BUV395 (564000, clone SK7), anti-CD3-PerCP (345766, clone SK7), anti-CD56-APC (341027, clone NCAM16.2), and anti-CD107a-PE (555801, clone H4A3) were procured from BD Biosciences. anti-CD3-pacific blue antibody (344823, clone SK7) was from Biolegend. L18-MDP (tlrl-lmdp) was from Invivogen. Agarose TUBE 2 (UM402) was purchased from Lifesensors. Lipofectamine™ 2000 (11668019) and Lipofectamine™ 3000 (L3000015) Transfection Reagents were procured from Thermo Fisher Scientific. IL-2 cytokine (2238131) was procured from Novartis.

### Cell culture and stimulation

Peripheral blood mononuclear cells (PBMC) were isolated using Ficoll gradient centrifugation. PBMCs were maintained for 2–3 h in serum free RPMI-1640 medium (Gibco, Life Technologies) and then stimulated with L18-MDP (10 µg/ml) or LPS (1 µg/ml). To enrich for human primary monocytes, PBMCs were cultured in Iscove's Modified Eagle's Medium (IMDM) (Gibco, Life Technologies) supplemented with 10% fetal bovine serum (FBS) and kept overnight at 37 °C. The day after, non-adherent cells were washed off. To measure intracytoplasmic TNF, PBMC-derived monocytes were stimulated by adding either 200 ng/ml L18-MDP or 200 ng/ml lipopolysaccharide (LPS) (Sigma-Aldrich) in the presence of Golgistop (BD Biosciences) for 2.5 h. K562 cells (ATCC; CCL-243) were cultured in RPMI-1640 medium supplemented with 1% L-glutamine, 10% v/v FBS, and 1% penicillin/streptomycin. Human embryonic kidney HEK293T cells (ATCC, CRL3216) and coloncacinoma HCT116 cells (ATCC, CCL247) were cultured in Dulbecco's Modified Eagle Medium (DMEM) medium (Thermo Fisher Scientific) supplemented with 1% L-glutamine, 10% v/v FBS, and 1% penicillin/streptomycin. For evaluating *IL8* transcriptional level, HCT116 cells stimulated with 1 µg/ml L18-MDP for 2, 4 and 8 h or with 5 µg/ml tunicamycin (Sigma-Aldrich) for 8 and 24 h. To analyze transcriptional level of *IL8*, *CXCL1*, *CXCL2* or *CHOP* in HCT116 cells 72 h post siRNA transfection, stimulation was performed with 1 µg/ml L18-MDP for 2, 4 and 8 h or with 5 µg/ml tunicamycin (Sigma-Aldrich) for 8 and 24 h.

### NK cells Degranulation assay

PBMCs were either directly cultured in complete RPMI-1640 medium supplemented with anti-CD107a alone or together with K562 to induce NK cells degranulation. To investigate degranulation in activated NK cells, PBMCs were incubated for 2 days in complete RPMI medium containing 600 U/ml IL-2 before co-culturing with K562 and anti-CD107a. Centrifugation was performed at 30 g, RT for 3 min and cells were incubated for 3 h at 37 °C. Surface staining was performed with anti-CD107a, anti-CD3, and anti-CD56. Flow cytometry was conducted on the FACS Canto II (BD Biosciences) and CD107a surface expression was investigated in the CD3^−^CD56^+^ cell population. Analysis was performed with Flowjo V9 software (TreeStar).

### Immunoblotting and silver staining

Cells were lysed in 1× cell lysis buffer (Cell Signaling Technologies) supplemented with 1 mM phenylmethylsulfonyl fluoride and 1× protease inhibitors. Normalization of protein concentration was performed by Bradford assay and equal amount of proteins were subjected to 10–12% SDS–PAGE followed by immunoblotting using different antibodies. Chemiluminescence signals were detected using the SuperSignal West Dura detection kit (Thermo Fisher Scientific) on the ChemiDoc^TM^ XRS + System (Bio-Rad) and analyzed with the ImageLab^TM^ software (Bio-Rad). SDS–PAGE silver staining was performed using silverQuest^TM^ (Invitrogen) according to the manufacturer’s protocols.

### Intracellular flow cytometry

Cells were washed with PBS and then fixed and permeabilized using the Cytofix/Cytoperm Kit (BD Bioscience) and stained with CD14, CD3 and TNF antibodies. Flow cytometry was performed on the LSRFortessa^TM^ flow cytometer (BD Biosciences) and analyzed with the Flowjo V9 software (TreeStar)^52^.

### Engineering of NOD2 knockout cell lines using CRISPR/Cas9 genome editing

The Alt-R® CRISPR-Cas9 (IDT technology) genome editing system was used according to the manufacturer’s instructions on HCT116 cells for the generation of knockouts. Electroporation was performed using the SE Cell Line 4D-Nucleofector® X Kit and the 4DNucleofector™ System (Lonza). Single cells were sorted into 96-well plates on a BD FACSAria (BD Bioscience) 48 h post transfection. In expanded clones, NOD2 knockout was functionally confirmed using NF-κB luciferase reporter gene assays.

### Quantitative real-time PCR analysis and ELISA

Total RNA was isolated using the RNeasy plus Kit (Qiagen) and reverse-transcribed to cDNA according to the manufacturer’s protocols (MultiScribe Reverse Transcriptase; Applied Biosystems). Relative transcriptional level were measured by SYBR Green dye-based quantitative real-time PCR (qRT–PCR) and analyzed using the ABI Prism 7500 Fast RT-PCR System (Applied Biosystems). GAPDH was used as a housekeeping marker. The list of primers is given in Supplementary Table 1. IL-8 secretion in the supernatant was quatified using the Human IL-8/CXCL8 DuoSet ELISA kit (R&D) and measured using a Synergy H1 microplate reader (BioTek Instruments) according to the manufacturer’s protocol.

### NF-κB luciferase reporter gene assays

HEK293T cells were transfected with the p55-A2-Luc luciferase reporter plasmid, internal control pTK-Green Renilla plasmid, NOD2 plasmids or control empty plasmids using Lipofectamine 2000^TM^ (Thermo Fisher Scientific) according to manufacturer’s recommendations. L18-MDP stimulation (200 ng/ml, Invivogen) was performed for 7 h followed by measurement of luciferase activity using the Dual Luciferase Assay Kit (Biotium). To study NF-kB activity in VCP knockdown HCT116 cells, cells were transfected with the p55-A2-Luc luciferase reporter plasmid and pTK-Green Renilla plasmid by lipofectamine 3000^TM^ (Thermo Fisher Scientific) 24 h after siRNA treatment. L18-MDP stimulation (200 ng/ml, Invivogen) was performed for 4 and 8 h after 72 h of siRNA transfection . To screen NOD2 KO HCT116, the NF-κB luciferase reporter assay was performed as described for HEK293T cells.

### Co-immunoprecipitation assays

HEK293T cells were transfected with 10 µg FLAG-NOD2 WT and mutants alone or together with RIPK2 using polyethyleneimine (PEI; Polysciences). After 72 h, the cells were washed in PBS and lysed in the RIPA buffer (10 mM Tris–HCl, pH 7.5, 150 mM NaCl, 5 mM EDTA, 1% NP-40, 10% glycerol) supplemented with 30 mM sodium pyrophosphate, 50 mM sodium fluoride, 1 mM phenylmethylsulfonyl fluoride and 1 × protease inhibitors (Roche). Immunoprecipitation was performed by incubating the lysates with anti-FLAG M2 Affinity Gel (Sigma-Aldrich) for 7 h at 4 °C on the rocker platform. Beads were washed three times in 1 ml ice-cold RIPA buffer and bound proteins were eluted by boiling the beads in gel loading buffer. HCT116 cells stably expressing NOD2 constructs were directly treated with L18-MDP for indicated time points and lysed in RIPA buffer.

### Purification of endogenous Ub conjugates

NOD2 KO HCT116 cells with WT or mutant NOD2 variants were stimulated with 200 ng/ml L18-MDP (Invivogen) for 1 and 2 h. Cells were washed in PBS and lysed in cell lysis buffer (50 mM Tris–HCl, pH 7.5, 150 mM NaCl, 1 mM EDTA, 1% NP-40, 10% glycerol) containing 1 mM phenylmethylsulfonyl fluorid, 1 × protease inhibitors (Roche) and 1 mM N-ethylmaleimide. Ubiquitinated proteins were isolated using Tandem Ubiquitin Binding Entities (TUBEs, LifeSensors) according to the manufacturer’s instructions. Eluted samples were analyzed by western blotting on 10% SDS-PAGE.

### siRNA transfection

Two siRNAs targeting human VCP and non-targeting (NT) siRNA oligonucleotides were designed and validated as described by Paola Magnaghi et al.^67^. HCT116 cells were transfected with 5 nM of siRNA oligonucleotides using Lipofectamine^TM^ 3000 (Thermo Fisher Scientific) according to manufacturer’s recommendations and incubated for 72 h. Knockdown efficiency was assessed by qPCR and western blotting. The sequences of oligonucleotides are provided in Supplementary Table 1.

### Analysis of cell death in HCT116 coloncarcinoma cell lines

NOD2 knockout and lentiviral reconstituted NOD2 WT HCT116 cells treated with siRNAs targeting human VCP and non-targeting (NT) siRNA oligonucleotides were stimulated with 5 µg/ml tunicamycin (Sigma-Aldrich) for 24 h. To measure cell death, HCT116 cells were stained with Annexin V and DAPI (Thermo Fisher Scientific) and analyzed by flow cytometry.

### Nano-LC MS/MS analysis

Samples were seperated by SDS-PAGE (SERVAGel TG PRiME 4–20%, Serva). Gels were Coomassie stained (Simply Blue, Expedeon) and the area containing proteins was excised. Gel slices were destained (50% acetonitril, 50 mM NH4HCO3) and subjected to in-gel digestion using the following steps: For protein reduction and alkylation, gel slices were first incubated in 45 mM DTE/50 mM NH4HCO3 for 30 min at 55 °C and then incubated for 30 min in 100 mM iodoacaetamide/50 mM NH4HCO3. In-gel digestion was done using 0.7 µg Trypsin at 37 °C overnight. Samples were analyzed by nano-LC MS/MS using an Ultimate 3000 nano liquid chromatography system (ThermoFisher Scientific) coupled to a TripleTOF 5600 + instrument (Sciex). As solvent A 0.1% formic acid and as solvent B acetonitrile with 0.1% formic acid was used. Peptides were separated at a flow rate of 200 nL/min on an Acclaim PepMap RSLC C18 column (75 μm × 50 cm, Thermo Fisher Scientific) with the following gradient: from 2% B to 25% B in 120 min followed by 25% B to 50% B in 10 min. For mass spectrometry, the ion source was operated at a needle voltage of 2.3 kV. Mass spectra were acquired in cycles of one MS scan from 400 m/z to 1250 m/z and up to 40 data dependent MS/MS scans of the most intensive peptide signals. For protein identification (FDR < 1%) and label free quantification, the MaxQuant software platform^68^ was used in combination with the Human subset of the UniProt database. The mass spectrometry proteomics data have been deposited to the ProteomeXchange Consortium via the PRIDE partner repository with the dataset identifier PXD031539^69^.

### Statistical analysis

Prism version 6 (GraphPad Software, USA) was used for statistical analysis of experimental data. Probability (P) values were calculated using two-way repeated-measures ANOVA and P values < 0.05 were considered to be statistically significant. Statistical details of experiments can be found in the figures and figure legends. Biologically independent experiments are referred to as n.

## Supplementary Information

Supplementary Information.

## Data Availability

The data generated in this study is available upon request, please contact the corresponding author.
