# Supplementary material for: Valosin-containing protein-regulated endoplasmic reticulum stress causes NOD2-dependent inflammatory responses
Source: Sci Rep. 2022 Mar 10;12:3906. doi: 10.1038/s41598-022-07804-1 (PMC8913691; doi:10.1038/s41598-022-07804-1)

Supplementary Information

Valosin-containing protein-regulated endoplasmic reticulum stress causes NOD2-dependent inflammatory responses

Maryam Ghalandary[1,#], Yue Li[1,#], Thomas Fröhlich[2], Thomas Magg[1], Yanshan Liu[1], Meino Rohlfs[1], Sebastian Hollizeck[1], Raffaele Conca[1], Tobias Schwerd[1], Holm H. Uhlig[3], Philip Bufler[1,4], Sibylle Koletzko[1,5], Aleixo M. Muise[6,7,8,9,10], Scott B. Snapper[8,11,12,13], Fabian Hauck[1], Christoph Klein[1,8], and Daniel Kotlarz[1,8] *

#These authors contributed equally to the paper

*Corresponding author: Daniel Kotlarz

E-mail: daniel.kotlarz@med.uni-muenchen.de

Supplementary Table 1. Related to Materials and Methods: Sequences of siRNAs, sgRNA targets and primers used.

Sequence of primers used (5'-3')	
GAPDH forward	TGATGACATCAAGAAGGTGGTGAAG
GAPDH reverse	TCCTTGGAGGCCATGTGGGCCAT
IL8 forward	ACTCCAAACCTTTCCACCCCAAAT
IL8 reverse	ACAACCCTCTGCACCCAGTTTT
VCP forward	ATCCGTGAATCCATCGAGAG
VCP reverse	GGAATCTGAAGCTGCCAAAG
CHOP forward	ATGAACGGCTCAAGCAGGAA
CHOP reverse	GGGAAAGGTGGGTAGTGTGG
CXCL1 forward	TCCAGAGCTTGAAGGTGTTGCC
CXCL1 reverse	AACCAAGGGAGCTTCAGGGTCA
CXCL2 forward	GGCAGAAAGCTTGTCTCAACCC
*CXCL*2 reverse	CTCCTTCAGGAACAGCCACCAA

Sequence of siRNAs and sgRNA (5'-3')	
VCP-si1	GAAUAGAGUUGUUCGGAAU
VCP-si2	GGAGGUAGAUAUUGGAAUU
Non-targeting (NT) siRNA	UGGUUUACAUGUCGACUAA
sg-NOD2-T1	CGGGACCTAACCAGACAAT
sg-NOD2-T2	GCTTCCTCAGTACCTATGA
sg-NOD2-T3	GTGCCAAAGGTGTCGTGCCA
sg-NOD2-T4	GAGGCCTGGATGCACATCGT

Figure S1. Impaired NK cells degranulation in patient. Representative FACS analysis of NK cell degranulation against K562 cells (n=2). Healthy donor (HD). Patient (Pat).

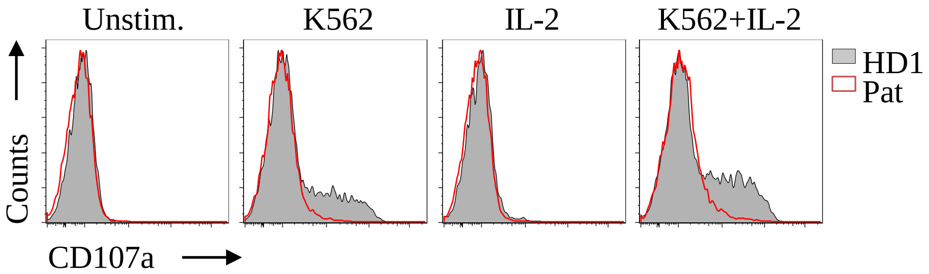

Figure S2. Immunoblotting showing the endogeneous interaction of VCP and NOD2 by immunoprecipitation with an antibody to VCP and co-immunoprecipitation of NOD2 in HCT-116 cells with or without KO of NOD2.

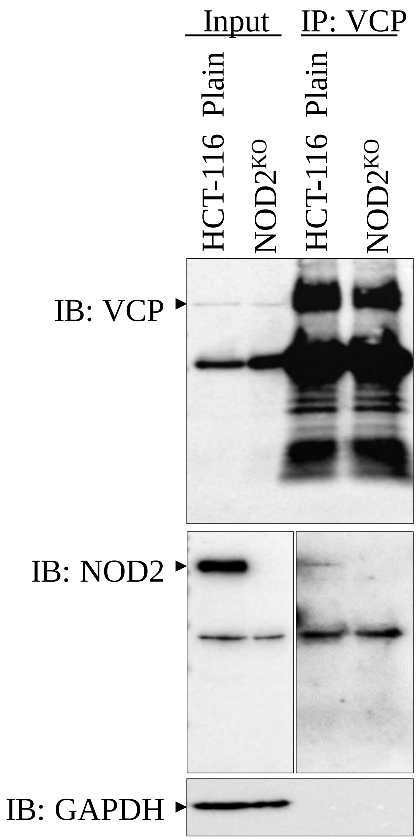

Figure S3. Immunoprecipitation of Flag-NOD2 upon stimulation with L18-MDP in HCT-116 cells with or without knockdown of VCP.

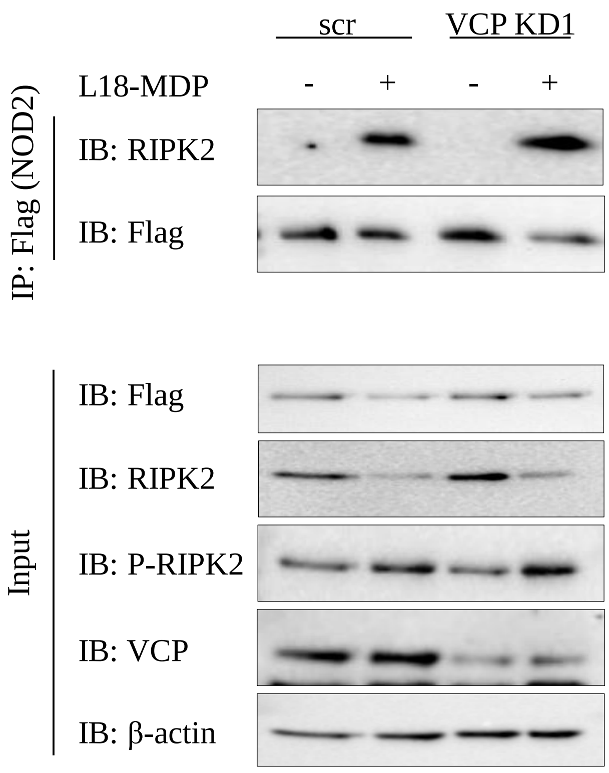

Figure S4. Related to Figure 4.B. Evaluation of VCP knock-down efficiency in HCT116 cells. Quantitative RT–PCR analysis to measure VCP transcriptional level in L18-MDP-treated HCT116 cells transfected with si-NT or si-VCP. Data represent mean ± SEM of three independent experiments.

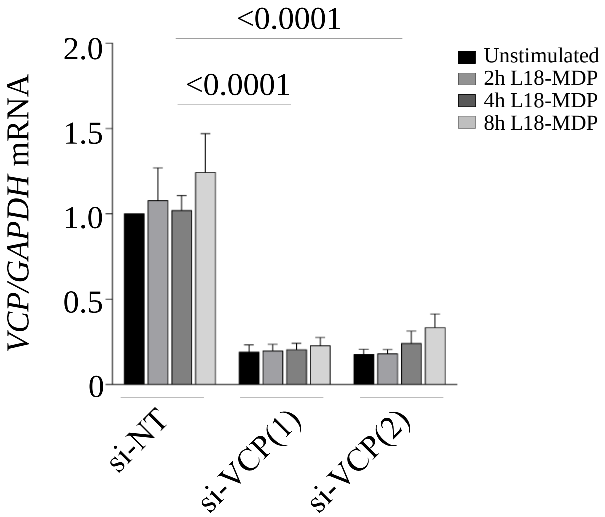

Figure S5. Immunoblotting showing the UPR pathway activity upon (A) tunicamycin and (B) thapsigargin stimulation in both WT HCT116 cells and NOD2 KO cells. Asterisk (*) indicates unspecific bands.

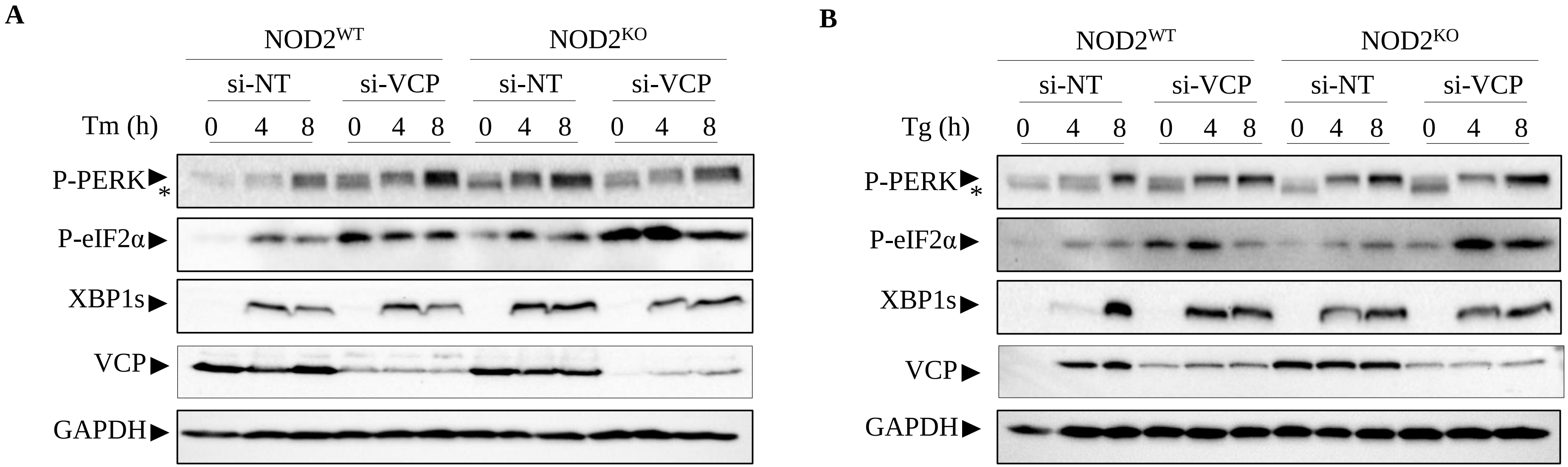

Figure S6. Related to Figure 4.C and D. Evaluation of cell death response upon tunicamycin stimulation on VCP knock-down HCT116 cells. Representative FACS analysis of Annexin V/DAPI staining (n=2) in NOD2 knock-out and lentiviral reconstituted NOD2 WT HCT116 cells transfected with si-NT or si-VCP upon tunicamycin stimulation.

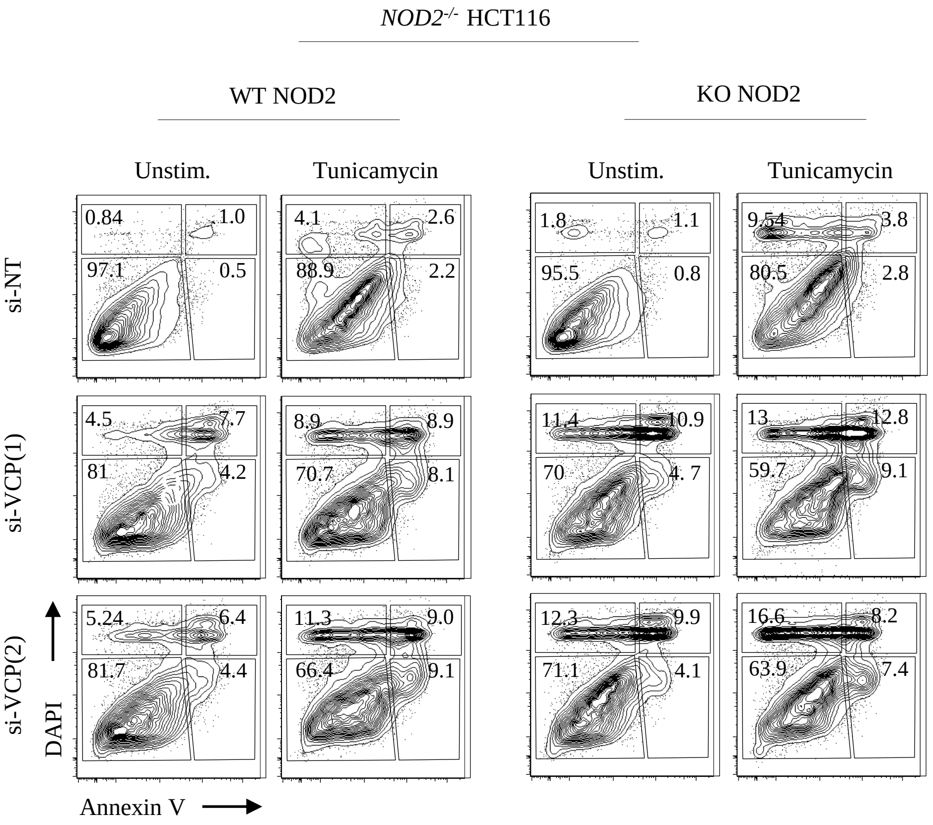

Figure S7. Uncropped image of Figure 3A.

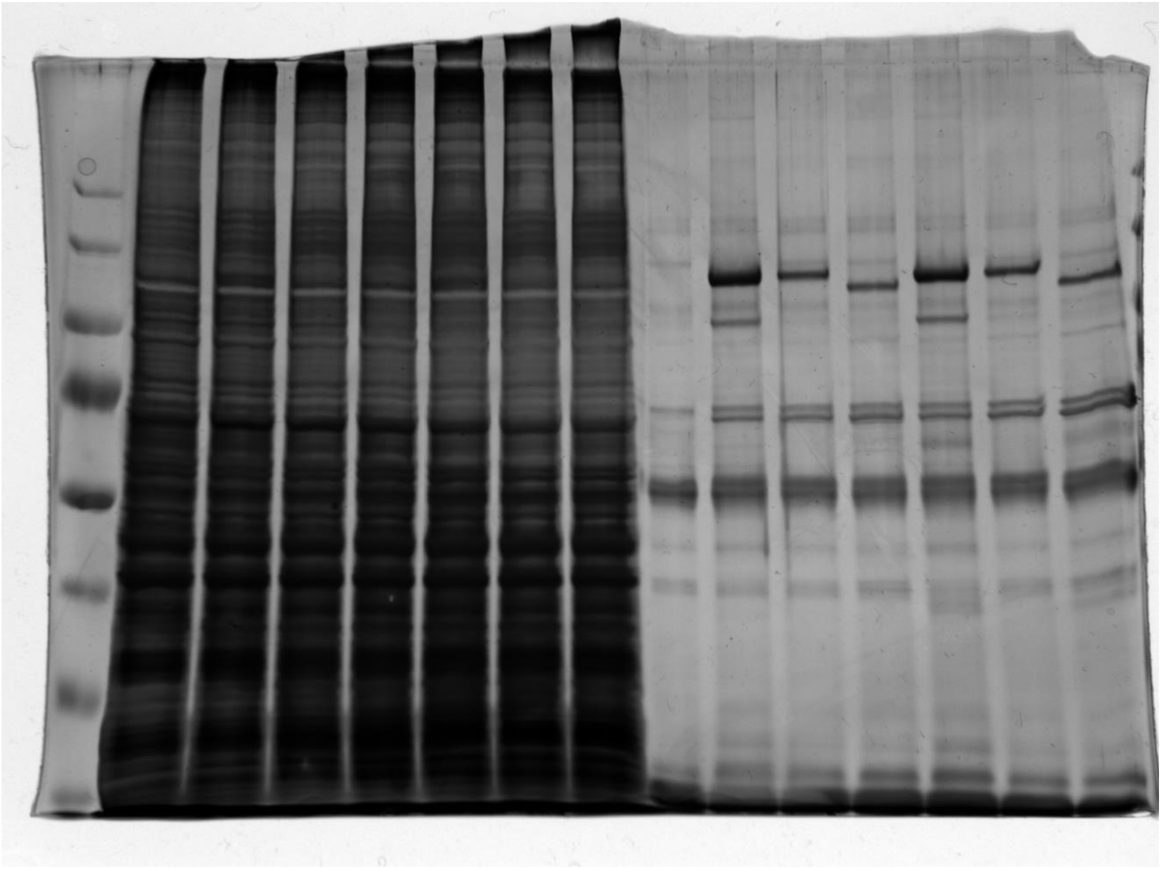

Figure S8. Uncropped images from immunoblots in Figure 1C. Membrane was cut at around 55 kDa after transfer and before hybridization with antibodies, which did not interfere with the detection of proteins with the size of 65 kDa, 38 kDa, 42/44 kDa, and 37 kDa. Due to limited access to patient material, the experiment was done once.

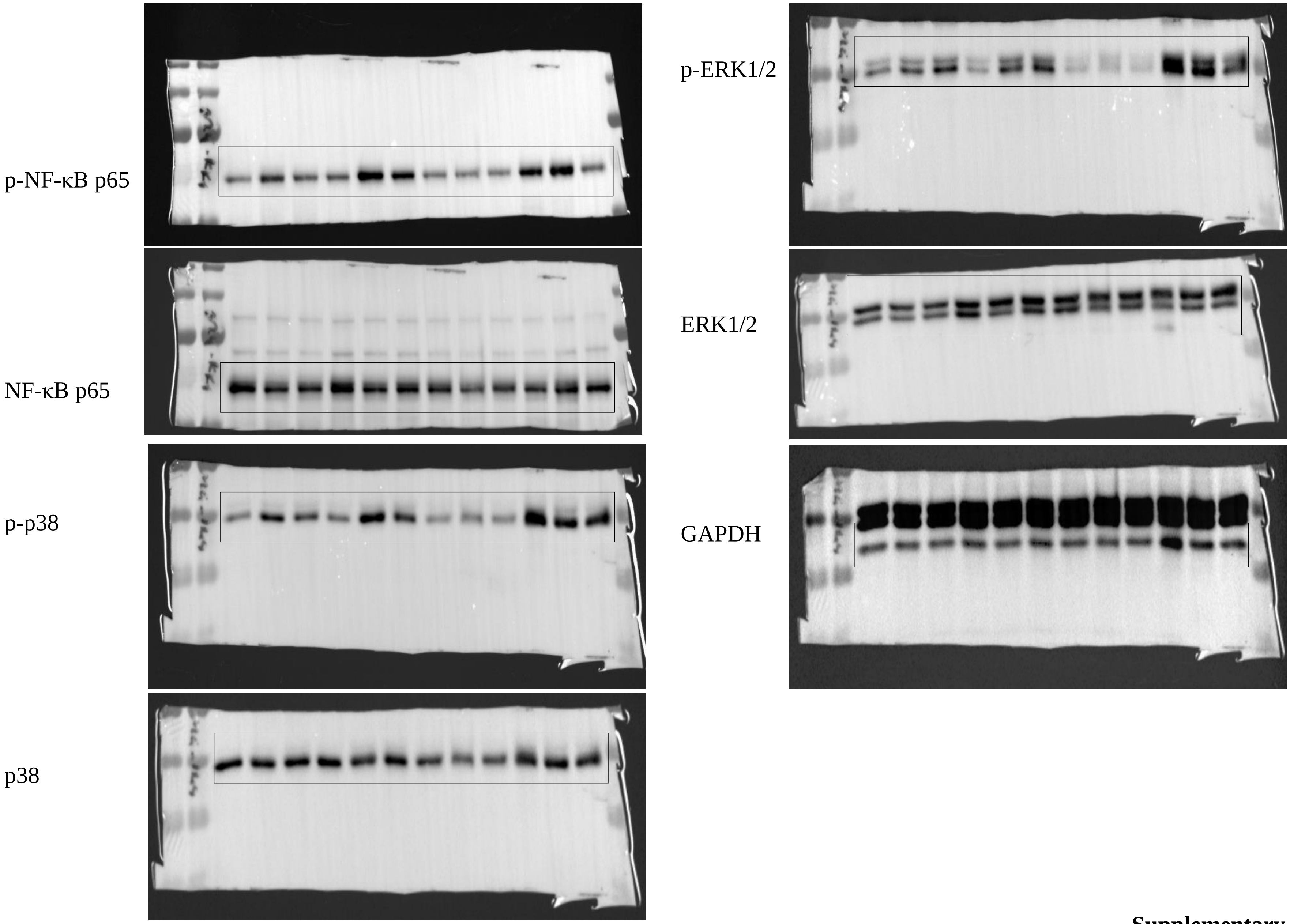

Supplementary Figure 8

Figure S9. Uncropped full length membrane images from immunoblots in Figure 2D.

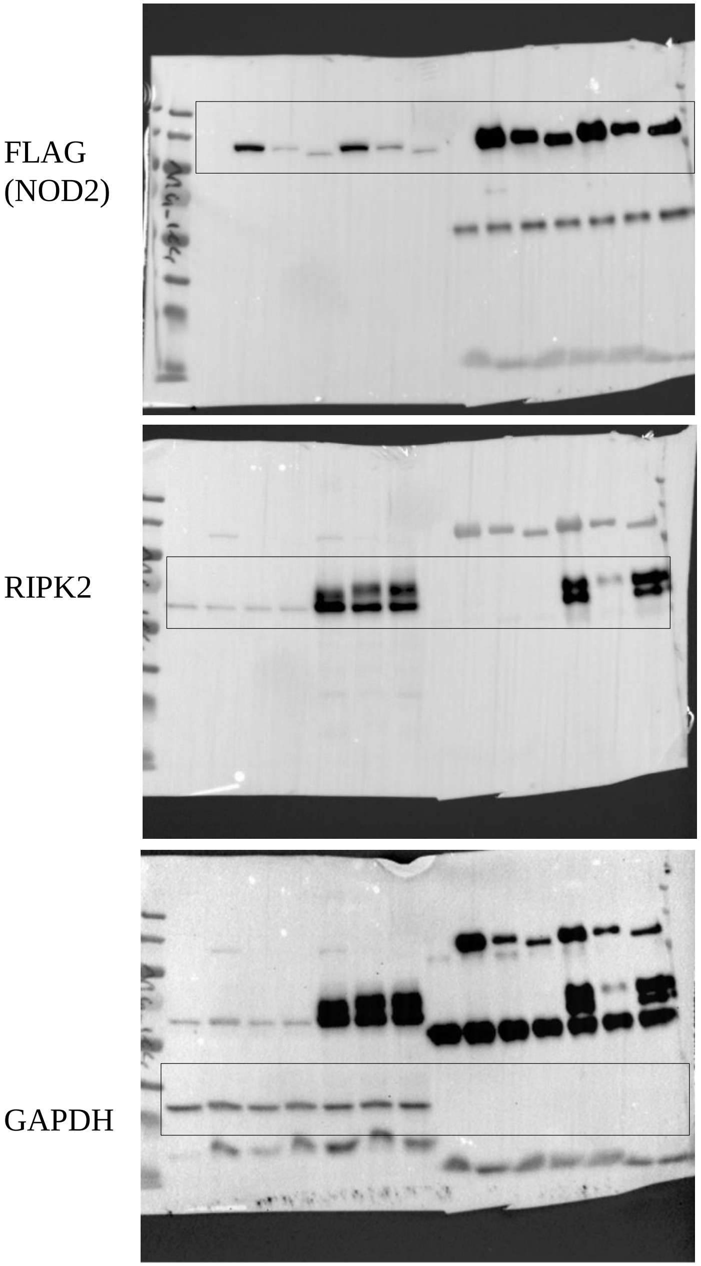

Supplementary Figure 9

Figure S10. Uncropped full length membrane images from immunoblots in Figure 2E.

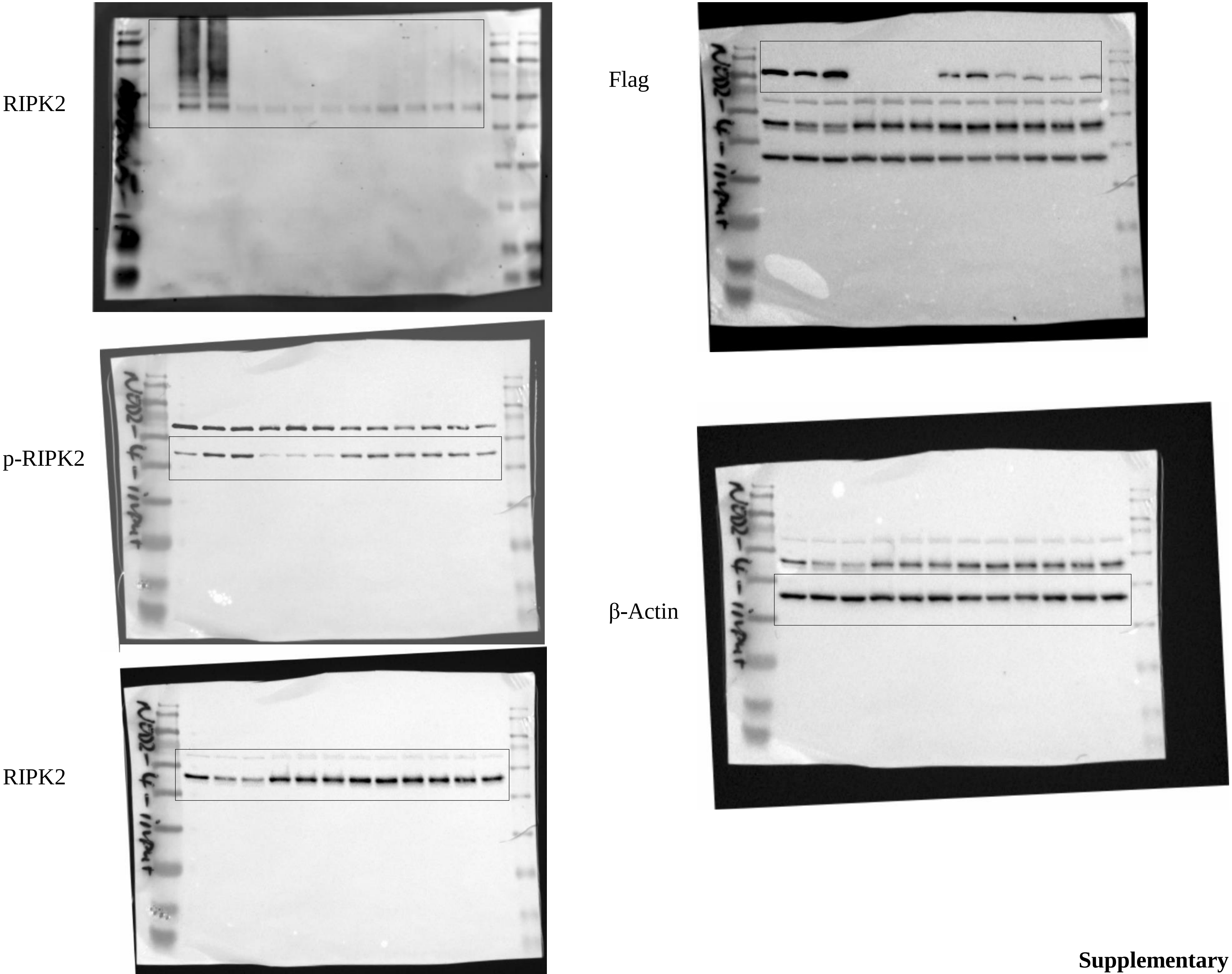

Supplementary Figure 10

Figure S11. Uncropped full length membrane images from immunoblots in Figure 3C.

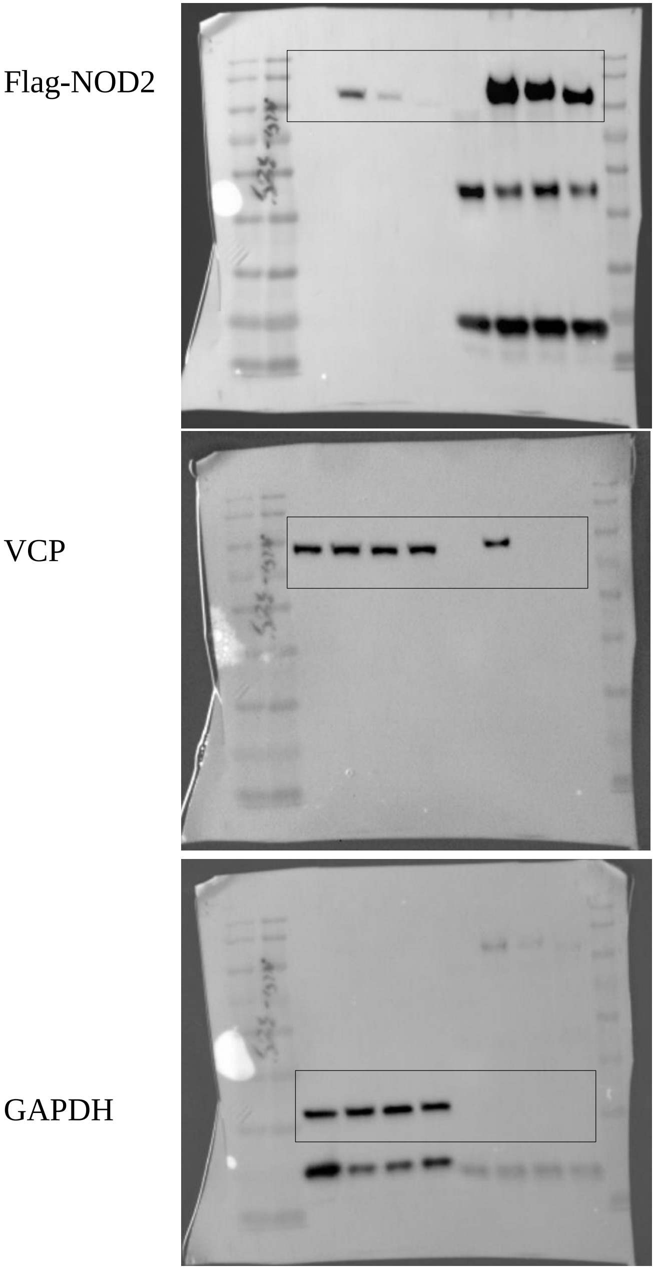

Supplement: Supplementary file 1 — Supplementary Information. [file 41598_2022_7804_MOESM1_ESM.pdf]
